# *NLRP3* Is Expressed in the Spiral Ganglion Neurons and Associated with Both Syndromic and Nonsyndromic Sensorineural Deafness

**DOI:** 10.1155/2016/3018132

**Published:** 2016-11-14

**Authors:** Penghui Chen, Longxia He, Xiuhong Pang, Xiaowen Wang, Tao Yang, Hao Wu

**Affiliations:** ^1^Department of Otolaryngology-Head and Neck Surgery, Xinhua Hospital, Shanghai Jiaotong University School of Medicine, Shanghai, China; ^2^Ear Institute, Shanghai Jiaotong University School of Medicine, Shanghai, China; ^3^Shanghai Key Laboratory of Translational Medicine on Ear and Nose Diseases, Shanghai, China; ^4^Department of Otorhinolaryngology-Head and Neck Surgery, Taizhou People's Hospital, Jiangsu Province, China; ^5^Department of Otorhinolaryngology-Head and Neck Surgery, Shanghai Ninth People's Hospital, Shanghai Jiaotong University School of Medicine, Shanghai, China

## Abstract

Nonsyndromic deafness is genetically heterogeneous but phenotypically similar among many cases. Though a variety of targeted next-generation sequencing (NGS) panels has been recently developed to facilitate genetic screening of nonsyndromic deafness, some syndromic deafness genes outside the panels may lead to clinical phenotypes similar to nonsyndromic deafness. In this study, we performed comprehensive genetic screening in a dominant family in which the proband was initially diagnosed with nonsyndromic deafness. No pathogenic mutation was identified by targeted NGS in 72 nonsyndromic and another 72 syndromic deafness genes. Whole exome sequencing, however, identified a p.E313K mutation in* NLRP3*, a gene reported to cause syndromic deafness Muckle-Wells Syndrome (MWS) but not included in any targeted NGS panels for deafness in previous reports. Follow-up clinical evaluation revealed only minor inflammatory symptoms in addition to deafness in six of the nine affected members, while the rest, three affected members, including the proband had no obvious MWS-related inflammatory symptoms. Immunostaining of the mouse cochlea showed a strong expression of NLRP3 in the spiral ganglion neurons. Our results suggested that* NLRP3* may have specific function in the spiral ganglion neurons and can be associated with both syndromic and nonsyndromic sensorineural deafness.

## 1. Introduction

Hearing loss is a common sensory deficit that is genetically heterogeneous. It is estimated that about 70% of all inherited deafness is nonsyndromic, while the remaining 30% is syndromic. To date, more than 100 loci for nonsyndromic deafness have been mapped (http://hereditaryhearingloss.org/) and more than 400 syndromes have been described in which deafness is part of the anomalies. In some cases, mutations in the same gene can cause both nonsyndromic and syndromic deafness. While the number of causative genes is usually limited for each individual case of syndromic deafness, nonsyndromic deafness is often phenotypically similar among cases of different molecular etiologies, rendering the genetic diagnosis difficult for this type of hearing loss.

The advent of next-generation sequencing (NGS) provided an ideal tool to answer this challenge, as combined technologies of targeted genomic enrichment and high-throughput sequencing make it possible to sequence over a hundred deafness genes simultaneously at a reasonable cost [1, 2]. In recent years, a variety of targeted NGS panels have been implemented in genetic screening of deafness. The number of targeted genes ranged from 50 to over 200, typically including all known nonsyndromic deafness genes up to the date of the design and differing in the number of syndromic deafness genes [3–8]. Though those panels theoretically should able to cover most genes associated with nonsyndromic deafness, it remains possible that some syndromic deafness genes outside the panels may lead to clinical phenotypes similar to nonsyndromic deafness.

In this study, we showed one such example in a dominant family segregated with apparently nonsyndromic deafness. After targeted NGS failed to identify any causative mutation in 144 known deafness genes, an E313K mutation in* NLRP3* was revealed by whole exome sequencing which has been previously linked to syndromic deafness Muckle-Wells Syndrome (MWS, OMIM # 191900) [9]. Reports on such cases may improve the precise genetic diagnosis of deafness.

## 2. Materials and Methods

### 2.1. Subjects

**Figure 1:**
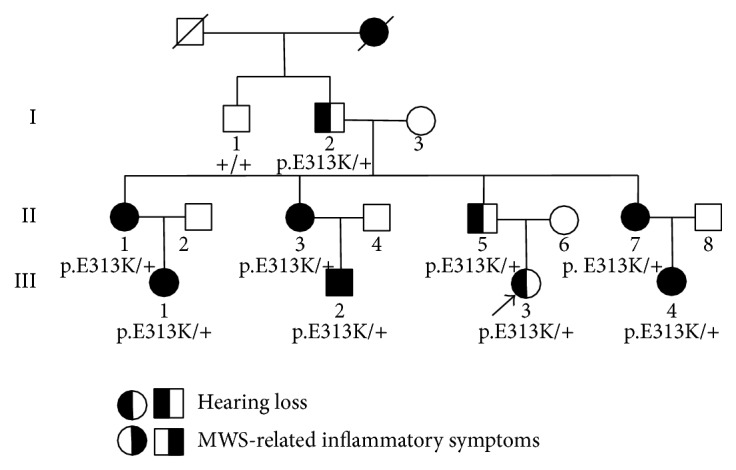
Pedigree of Family C277 with progressive hearing loss and MWS-related inflammatory symptoms. The genotype of the p.E313K mutation in* NLRP3* was marked under the family members. Proband III3 was pointed by an arrow.

A Chinese Han family (Family C277) segregated with autosomal dominant hearing loss was recruited through Xinhua Hospital, Shanghai, China. As shown in Figure 1, this family consisted of 9 affected family members and 1 unaffected family member. All affected members had bilateral, late-onset sensorineural hearing loss. The family members gave written, informed consent to participate in the present study. This study was approved by the ethics committee of Xinhua Hospital, Shanghai Jiaotong University School of Medicine.

### 2.2. Clinical Characterization

The hearing levels of all affected family members were measured by air and bone conducted pure tone audiometry. After detection of the p.E313K mutation in* NLRP3*, the affected family members received a follow-up clinical evaluation focusing on the MWS-related inflammatory symptoms including chronic fatigue, recurrent fever, headache, ocular symptoms such as conjunctivitis, uveitis, papillary edema, and opticus neuritis, oral ulcers, abdominal pain, proteinuria, musculoskeletal symptoms such as arthralgia, arthritis, and myalgia, and skin symptoms such as erythematous rash and cold-induced urticaria.

### 2.3. Targeted NGS and Whole Exome Sequencing

Genomic DNAs from the 10 family members were extracted from whole blood using Blood DNA kit (TIANGEN Biotech, Beijing, China). Targeted NGS of 144 known deafness genes (see complete list of the genes in Supplementary Table  S1 in Supplementary Material available online at http://dx.doi.org/10.1155/2016/3018132) was performed in proband III-3 using the MyGenotics gene enrichment system (MyGenotics, Boston, MD, USA) and the Illumina HiSeq 2000 sequencer (Illumina, San Diego, CA, USA) as previously described [10]. Whole exome sequencing was performed in family members I2, III2, III4, and I1 using the Agilent SureSelect V5+UTR Exome Enrichment Kit (Agilent, Santa Clara, CA, USA) and the Hiseq X Ten sequencer (Illumina, San Diego, CA, USA) as previously reported [11]. The reads were aligned to HG19 using the BWA software and the variants were called using the Genome Analysis Toolkit (GATK), both with the default parameters. SNVs and indels were presented using Variant Call Format (VCF) version 4.1 and annotated using the ANNOVAR software. To identify the candidate pathogenic mutations, exclusive filtering criteria were applied to the variants including the following: (1) SNVs in nonsplicing region leading to synonymous amino acids; (2) SNVs and indels called in off-target regions; (3) variants with maximum minor allele frequency (MAF) greater than 0.001 in public databases 1000 Genomes Project, NHLBI Exome Sequencing Project (ESP) and the Examination for Architects in Canada (ExAC).

### 2.4. Immunostaining of the Mouse Cochlea

Immunostaining of the mouse cochlea was performed as previously described [11]. Briefly, P60 mouse cochlea was extracted, perfused, and fixed in PFA overnight. The cochlea was then washed in 0.1 M PBS, incubated in 5% EDTA for 5 days, dehydrated in 30% sucrose, embedded in OCT, and frozen at −20°C. Frozen sections (10 *μ*m) were obtained parallel to the modiolus. The slides were rewarmed at room temperature for 30 minutes, washed in PBS, incubated with a blocking buffer containing 5% donkey serum, 0.3% Triton X-100, and 1% BSA in PBS for 1 hour at room temperature, incubated in the 1 : 500 mouse anti-TUJ1 (MMS435P-250, Covance, Princeton, USA) and 1 : 200 rat anti-NLRP3 (MAB7578-SP, RnD, Minneapolis, USA) primary antibodies at 4°C overnight, and detected with 1 : 500 secondary antibodies conjugated to Alexa 488 and Alexa 594 (Jackson Immunoresearch, West Grove, USA). The slides were mounted in Prolong-Gold Antifade reagent with DAPI (Invitrogen, Carlsbad, USA) and examined with confocal fluorescence microscopy (LSM710, Zeiss, Berlin, Germany).

## 3. Results

### 3.1. Auditory Characteristics

**Figure 2:**
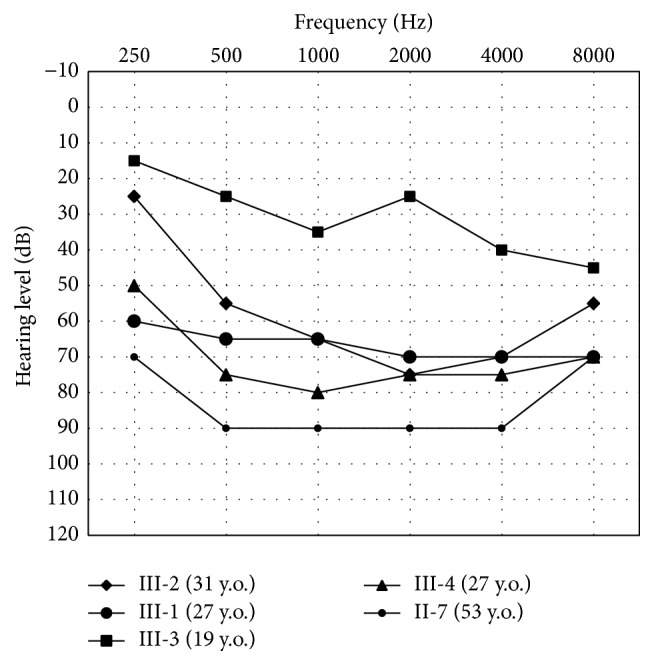
Audiometric features of affected individuals III1, III2, III3, III4, and II7 at age of 19–53 years. Pure tone hearing thresholds were shown as the averages of the left and right sides. The rest of the four affected individuals I2 (80 y.o.), II1 (56 y.o.), II3 (55 y.o.), and II5 (59 y.o.) had hearing thresholds of 90 dB or higher.

The affected members in Family C277 exhibited bilateral, late-onset, slowly progressive hearing impairment (Figure 2). The age at onset was around 20 years. The hearing impairment began in the middle and high frequencies, gradually progressed to all frequencies, and eventually reached profound in the sixth decade. There was no evidence of vestibular dysfunction in any member. Initial clinical evaluation of the proband III3 revealed no significant abnormalities other than the hearing impairment.

### 3.2. Exclusion of 144 Known Deafness Genes

To identify the genetic cause of the deafness in Family C277, we first screened 144 known deafness genes in proband III3 including 72 nonsyndromic and 72 additional syndromic deafness genes. Targeted NGS generated 2514581 mapped reads with an averaged on-target sequencing depth of 299x. 92.4% of the targeted regions were covered with at least 10x in depth. A total of four heterozygous nonsynonymous candidate variants with MAF of 0.01 or less were identified including p.Q1495fs in* PCDH15* (NM_001142767), p.M1209I in* COL4A3* (NM_000091), p.G204S in* EDN3* (NM_207033), and p.R36H in* KCNE1* (NM_001127670). Sanger sequencing in all 10 family members, however, revealed that none of the four variants segregated with the hearing loss phenotype in Family C277.

### 3.3. Identification of the p.E313K Mutation in* NLRP3*

**Figure 3:**
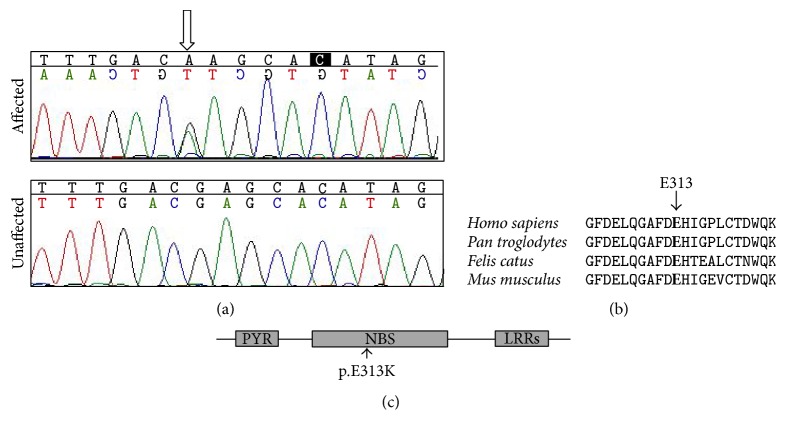
The p.E313K (c.G937A) mutation of* NLRP3*. (a) Chromatograms of the mutant and wild-type sequences. (b) Conservation of the E313 residue of NLRP3 in* Homo sapiens, Pan troglodytes*,* Felis catus*, and* Mus musculus*. (c) Domain structure of NLRP3 protein with the location of the p.E313K mutation marked by an arrow.

To further investigate the genetic cause of the deafness in Family C277, we performed whole exome sequencing in affected family members I2, III2, and III3 and unaffected family member I1. The mean sequencing depth for I2, III2, III3, and I1 was 76.67x, 93.72x, 91.05x, and 98.94x, respectively. An averaged 98% of the targeted region was covered by at least 5x in depth. A total of seven nonsynonymous candidate variants with MAF of 0.01 or less were identified in affected individuals I2, III2, and III3 but not in unaffected individual I1. Sanger sequencing in all 10 family members revealed a p.E313K (NM_001127462: c.G937A) mutation in* NLRP3*, a gene not included in any of the targeted NGS panels for deafness in previous reports (see Section 4 for details), as the only pathogenic mutation segregating with the hearing loss phenotype. The p.E313K mutation is located in the NBS domain of NLRP3 and changed an evolutionary conserved amino acid (Figure 3). This mutation was predicted as pathogenic by computer programs Mutation Taster, SIFT, and PROVEAN (prediction scores of 0.794, 2.80, and −3.02, resp.). It was not seen in 300 Chinese Han normal hearing controls.

### 3.4. Characteristics of the Autoinflammatory Abnormalities

The p.E313K mutation in* NLRP3* has been previously reported to be associated with inherited autoinflammatory disease Muckle-Wells Syndrome (MWS) [9]. A follow-up clinical evaluation therefore was performed in all 9 affected family members focusing on the MWS-related autoinflammatory features (Table 1). Different from the previous report, in six of the nine affected members only minor inflammatory symptoms were present including conjunctivitis and uveitis (*n* = 4), oral ulcers (*n* = 3), arthralgias and arthritis (*n* = 1), and erythematous rash (*n* = 2). The rest three affected members I2, II5, and III3 had no obvious MWS-related inflammatory symptoms. None of the nine affected members had chronic fatigue, recurrent fever, headache, pericarditis, abdominal pain, and proteinuria. The occurrence of the MWS-related inflammatory symptoms was apparently not associated with age. The affected individual I2 was 80 years old but did not have any inflammatory symptoms. On the contrary, the affected individual III-4 was only 27 years old but showed conjunctivitis, uveitis, and erythematous rash at a quite early age.

### 3.5. Expression of NLRP3 in the Spiral Ganglion Neurons

**Figure 4:**
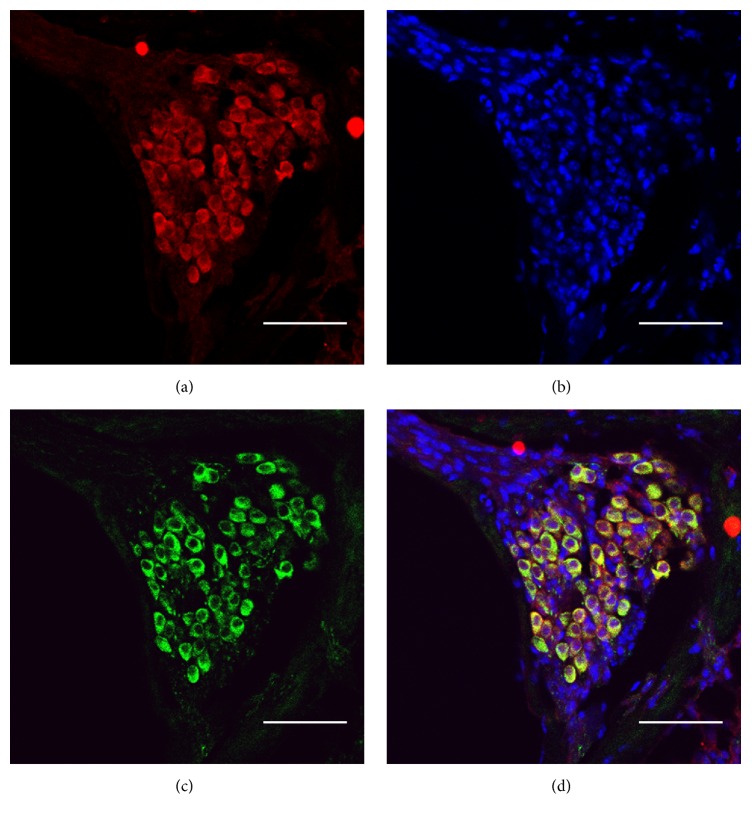
Expression of NLRP3 in the spiral ganglion neurons of the P60 mouse cochlea. ((a)–(c)) NLRP3, cell nucleus, and Tuj1 were stained as red, blue, and green, respectively. (d) Colocalization of NLRP3 and Tuj1 staining. Bars: 50 *μ*m.

Immunostaining showed that NLRP3 was strongly expressed in the spiral ganglion neurons of adult (P60) mouse cochlea (Figure 4). Its expression was mainly distributed in the cytoplasm similar to the neural marker Tuj1.

## 4. Discussion

In this study, we identified a p.E313K mutation in* NLRP3* as the pathogenic cause of the deafness in Family C277.* NLRP3* encodes a pyrin-like protein expressed in innate immune cells such as neutrophils, monocytes, and dendritic cells [12]. The NLRP3 protein contains a pyrin domain, a nucleotide-binding site (NBS) domain where the E313 amino acid residue resides, and a leucine-rich repeat (LRR) motif (Figure 3(c)). It plays an important role in inflammation, immune response, and apoptosis, while the neurological function of NLRP3 was only implicated in the sensorineural hearing loss [13]. Dominant mutations in* NLRP3* may lead to a spectrum of inflammatory diseases including familial cold autoinflammatory syndrome (FCAS, OMIM # 120100), Muckle-Wells Syndrome (MWS, OMIM # 191900), and chronic infantile neurological cutaneous and articular syndrome (CINCA, OMIM # 607115).

The p.E313K mutation in* NLRP3* has been previously reported to be associated with MWS [9]. MWS is a rare autosomal dominant disorder characterized by episodic skin rash, arthralgias, recurrent fever, and renal amyloidosis as well as late-onset sensorineural hearing loss. In a large dominant family of European descent in the report [12], a majority or all of the thirteen affected family members with the p.E313K mutation in* NLRP3* had MWS-related inflammatory symptoms including chronic fatigue (100%), recurrent fever (31%), headache (54%), ocular symptoms such as conjunctivitis (85%) and uveitis (77%), oral ulcers (46%), pericarditis (23%), abdominal pain (31%), renal amyloidosis (77%), musculoskeletal symptoms such as arthralgias (85%), arthritis (69%), and myalgia (54%), and erythematous rash (54%). On the contrary, Family C277 in our study exhibited a much milder degree of MWS-related inflammatory symptoms, in which only 44% of the nine affected members had conjunctivitis and uveitis, 33% with oral ulcers, 18% with erythematous rash, and 11% with arthralgias and arthritis (Table 1). Chronic fatigue, recurrent fever, headache, pericarditis, abdominal pain, renal amyloidosis, or myalgia was not reported in any of them. Three affected members, I2, II5, and III3, were free of MWS-related inflammatory symptoms other than the hearing loss (i.e., nonsyndromic deafness). The hearing loss in Family C277, therefore, should be regarded as a mixed syndromic (II1, II2, II7, III1, III2, and III4) and nonsyndromic (I2, II5, and III3) deafness depending on the specific affected individuals. The key symptom in this family overall, however, should be the hearing loss as the inflammatory symptoms were either relatively minor or completely absent. We speculated that the complicate genotype-phenotype correlation of the p.E313K mutation in NLRP3 can be influenced by both genetic and environmental factors. The different genetic background of the ethnicity may explain the phenotypic difference observed between the Chinese and European families with the same mutation. The environmental factors may explain the intrafamilial difference observed in Family C277.

Due to the mild inflammatory symptoms, the disorder of Family C277 was indeed initially diagnosed as nonsyndromic deafness. To our knowledge, however,* NLRP3* was not included in any of the major targeted NGS panels for deafness in the previous and current studies (Supplementary Table  S2). The p.E313K pathogenic mutation, therefore, was not identified until whole exome sequencing was performed. On the other hand, our immunostaining results showed that NLRP3 is strongly expressed in the spiral ganglion neurons of mouse cochlea (Figure 4), implicating a specific role of* NLRP3* in the inner ear function. Previous reports showed that early treatment with IL-1 inhibitors Anakinra or Canakinumab in patients with* NLRP3* mutations can improve hearing and prevent irreversible renal damage from amyloidosis [12, 14]. The early genetic diagnosis of* NLRP3* mutations, therefore, is essential for proper clinical intervention. Our study suggested that syndromic deafness genes such as* NLRP3* should be recognized and targeted for genetic screening of nonsyndromic deafness by targeted NGS.

## 5. Conclusions

*NLRP3* may have specific function in the spiral ganglion neurons of the cochlea. Mutations in* NLRP3* can be associated with both syndromic and nonsyndromic sensorineural deafness. Such genes should be recognized and targeted for genetic screening of nonsyndromic deafness.

## Supplementary Material

The Supplementary Material includes Supplementary Table S1 - 144 genes targeted for the next-generation sequencing and Supplementary Table S2 - Representative targeted NGS panels for deafness in previous reports.

## Figures and Tables

**Table 1:** Clinical features of the patients with the p.E313K mutation in *NLRP3.*

Clinical symptoms	I2 (80)^*∗*^	II1 (56)^*∗*^	II3 (55)^*∗*^	II5 (59)^*∗*^	II7 (53)^*∗*^	III1 (27)^*∗*^	III2 (31)^*∗*^	III3 (19)^*∗*^	III4 (27)^*∗*^	% in the current study	% in the referenced study [9]
Chronic fatigue	—	—	—	—	—	—	—	—	—	0	100
Recurrent fever	—	—	—	—	—	—	—	—	—	0	31
Headache	—	—	—	—	—	—	—	—	—	0	54
Ocular symptoms											
Conjunctivitis	—	Y	—	—	Y	Y	—	—	Y	44	85
Uveitis	—	Y	—	—	Y	Y	—	—	Y	44	77
Papillary edema	—	—	—	—	—	—	—	—	—	0	15
Hearing loss	Y	Y	Y	Y	Y	Y	Y	Y	Y	100	92
Oral ulcers	—	Y	Y	—	—	—	Y	—	—	33	46
Pericarditis	—	—	—	—	—	—	—	—	—	0	23
Abdominal pain	—	—	—	—	—	—	—	—	—	0	31
Renal amyloidosis	—	—	—	—	—	—	—	—	—	0	77
Musculoskeletal symptoms											
Arthralgias	—	—	Y	—	—	—	—	—	—	11	85
Arthritis	—	—	Y	—	—	—	—	—	—	11	69
Myalgias	—	—	—	—	—	—	—	—	—	0	54
Skin symptoms											
Erythematous rash	—	—	—	—	Y	—	—	—	Y	18	54

^*∗*^Age (years) when tested.
