# Supplementary material for: NLRP3 Is Expressed in the Spiral Ganglion Neurons and Associated with Both Syndromic and Nonsyndromic Sensorineural Deafness
Source: Neural Plast. 2016 Nov 14;2016:3018132. doi: 10.1155/2016/3018132 (PMC5124661; doi:10.1155/2016/3018132)
Supplement: Supplementary file 1 — The Supplementary Material includes Supplementary Table S1 - 144 genes targeted for the next-generation sequencing and Supplementary Table S2 - Representative targeted NGS panels for deafness in previous reports. [file 3018132.f1.docx]

**Supplementary Table S1.** 144 genes targeted for the next-generation sequencing.

| [ACTG1](http://www.ncbi.nlm.nih.gov/omim/102560)^N^ | [COL9A1](http://www.ncbi.nlm.nih.gov/omim/120210)^S^ | FGF8^S^ | [HSD17B4](http://omim.org/entry/601860)^S^ | [MYO15A](http://www.ncbi.nlm.nih.gov/omim/602666)^N^ | PROKR2^S^ | [TBC1D24](http://www.omim.org/entry/613577)^N/S^ |
| --- | --- | --- | --- | --- | --- | --- |
| ALX3^S^ | [COL9A2](http://omim.org/entry/120260)^S^ | FGFR1^S^ | IL13^S^ | [MYO1A](http://www.ncbi.nlm.nih.gov/omim/601478)^N^ | [PRPS1](http://www.ncbi.nlm.nih.gov/omim/311850)^S^ | [TCOF1](http://www.ncbi.nlm.nih.gov/omim/606847)^S^ |
| [BSND](http://www.ncbi.nlm.nih.gov/omim/606412)^S^ | COMT2^S^ | FGFR3^S^ | [ILDR1](http://www.ncbi.nlm.nih.gov/omim/609739)^N^ | [MYO3A](http://www.ncbi.nlm.nih.gov/omim/606808)^N^ | [PTPRQ](http://omim.org/entry/603317)^N^ | [TECTA](http://www.ncbi.nlm.nih.gov/omim/602574)^N^ |
| CABP2^N^ | [CRYM](http://www.ncbi.nlm.nih.gov/omim/123740)^N^ | FLNA^S^ | [KARS](http://omim.org/entry/601421)^N/S^ | [MYO6](http://www.ncbi.nlm.nih.gov/omim/600970)^N^ | [RDX](http://www.ncbi.nlm.nih.gov/omim/179410)^N^ | TIMM8A^S^ |
| [CCDC50](http://www.ncbi.nlm.nih.gov/omim/611051)^N^ | [DFNA5](http://www.ncbi.nlm.nih.gov/omim/600994)^N^ | [FOXI1](http://www.ncbi.nlm.nih.gov/omim/601093)^S^ | [KCNE1](http://www.ncbi.nlm.nih.gov/omim/176261)^S^ | [MYO7A](http://www.ncbi.nlm.nih.gov/omim/276903)^N/S^ | RPGR^S^ | [TJP2](http://www.ncbi.nlm.nih.gov/omim/607709)^N^ |
| [CDH23](http://www.ncbi.nlm.nih.gov/omim/605516)^N/S^ | DFNB31^N/S^ | FREM1^S^ | [KCNJ10](http://omim.org/entry/602208)^N/S^ | [NDP](http://www.ncbi.nlm.nih.gov/omim/300658)^S^ | SALL1^S^ | [TMC1](http://www.ncbi.nlm.nih.gov/omim/606706)^N^ |
| [CEACAM16](http://www.ncbi.nlm.nih.gov/pubmed/21368133)^N^ | DFNB59^N^ | GATA3^S^ | [KCNQ1](http://www.ncbi.nlm.nih.gov/omim/607542)^S^ | NF2^N^ | SALL4^S^ | [TMIE](http://www.ncbi.nlm.nih.gov/omim/607237)^N^ |
| CHD7^S^ | DIABLO^N^ | GIPC3^N^ | [KCNQ4](http://www.ncbi.nlm.nih.gov/omim/603537)^S^ | [OTOA](http://www.ncbi.nlm.nih.gov/omim/607038)^N^ | [SANS](http://www.ncbi.nlm.nih.gov/omim/607696)^S^ | [TMPRSS3](http://www.ncbi.nlm.nih.gov/omim/605511)^N^ |
| [CIB2](http://omim.org/entry/605564)^N/S^ | [DIAPH1](http://www.ncbi.nlm.nih.gov/omim/602121)^N^ | [GJB2](http://www.ncbi.nlm.nih.gov/omim/121011)^N/S^ | KRT9^S^ | [OTOF](http://www.ncbi.nlm.nih.gov/omim/603681)^N^ | SEC23A^S^ | [TNC](http://www.omim.org/entry/187380)^N^ |
| [CLDN14](http://www.ncbi.nlm.nih.gov/omim/605608)^N^ | DIAPH3^N^ | GJB3^N/S^ | LAMA3^S^ | [P2RX2](http://www.omim.org/entry/600844)^N^ | [SEMA3E](http://www.omim.org/entry/608166)^S^ | [TPRN](http://www.ncbi.nlm.nih.gov/omim/613354)^N^ |
| CLPP^S^ | [DSPP](http://www.ncbi.nlm.nih.gov/omim/125485)^S^ | [GJB6](http://www.ncbi.nlm.nih.gov/omim/604418)^N^ | [LARS2](http://omim.org/entry/604544)^S^ | PABPN1^S^ | [SERPINB6](http://omim.org/entry/173321)^N^ | [TRIOBP](http://www.ncbi.nlm.nih.gov/omim/609761)^N^ |
| [CLRN1](http://www.ncbi.nlm.nih.gov/omim/606397)^S^ | ECM1^U^ | GPR98^S^ | [LHFPL5](http://www.ncbi.nlm.nih.gov/omim/609427)^N^ | [PAX3](http://www.ncbi.nlm.nih.gov/omim/606597)^S^ | [SIX1](http://www.omim.org/entry/601205)^N/S^ | TRMU^S^ |
| [COCH](http://www.ncbi.nlm.nih.gov/omim/603196)^N/S^ | [EDN3](http://www.ncbi.nlm.nih.gov/omim/131242)^S^ | [GPSM2](http://omim.org/entry/609245)^S^ | [LOXHD1](http://www.ncbi.nlm.nih.gov/omim/613072)^N^ | [PCDH15](http://www.ncbi.nlm.nih.gov/omim/605514)^N^/^S^ | [SIX5](http://www.ncbi.nlm.nih.gov/omim/600963)^S^ | [TSPEAR](http://www.omim.org/entry/612920)^N^ |
| [COL11A1](http://www.ncbi.nlm.nih.gov/omim/120280)^N/S^ | [EDNRB](http://www.ncbi.nlm.nih.gov/omim/131244)^S^ | GRHL2^N^ | LRTOMT^N^ | [PDZD7](http://www.ncbi.nlm.nih.gov/omim/612971)^S^ | [SLC17A8](http://omim.org/entry/607557)^N^ | [USH1C](http://www.ncbi.nlm.nih.gov/omim/605242)^S^ |
| [COL11A2](http://www.ncbi.nlm.nih.gov/omim/120290)^N/S^ | [ELMOD3](http://www.omim.org/entry/615427)^N^ | [GRXCR1](http://www.ncbi.nlm.nih.gov/omim/613283)^N^ | [MARVELD2](http://www.ncbi.nlm.nih.gov/omim/610572)^N^ | [PNPT1](http://omim.org/entry/610316?search=PNPT1&highlight=pnpt1)^N^ | [SLC26A4](http://www.ncbi.nlm.nih.gov/omim/605646)^N/S^ | USH1G^S^ |
| [COL2A1](http://www.ncbi.nlm.nih.gov/omim/120140)^S^ | [ESPN](http://www.ncbi.nlm.nih.gov/omim/606351)^N^ | HARS^S^ | MIR96^N^ | [POLR1C](http://www.omim.org/entry/610060)^S^ | [SLC26A5](http://www.ncbi.nlm.nih.gov/omim/604943)^N^ | [USH2A](http://www.ncbi.nlm.nih.gov/omim/276901)^S^ |
| [COL4A3](http://www.ncbi.nlm.nih.gov/omim/120070)^S^ | [ESRRB](http://www.ncbi.nlm.nih.gov/omim/602167)^N^ | [HARS2](http://omim.org/entry/600783)^S^ | [MITF](http://www.ncbi.nlm.nih.gov/omim/156845)^S^ | [POLR1D](http://www.omim.org/entry/613715)^S^ | [SMPX](http://omim.org/entry/300226)^N/S^ | [WFS1](http://www.ncbi.nlm.nih.gov/omim/606201)^N/S^ |
| [COL4A4](http://www.ncbi.nlm.nih.gov/omim/120131)^S^ | [EYA1](http://www.ncbi.nlm.nih.gov/omim/601653)^S^ | [HGF](http://www.ncbi.nlm.nih.gov/omim/142409)^N^ | [MSRB3](http://www.ncbi.nlm.nih.gov/omim/613719)^N^ | [POU3F4](http://www.ncbi.nlm.nih.gov/omim/300039)^N^ | [SNAI2](http://www.ncbi.nlm.nih.gov/omim/602150)^S^ | WHRN^N/S^ |
| [COL4A5](http://www.ncbi.nlm.nih.gov/omim/303630)^S^ | [EYA4](http://www.ncbi.nlm.nih.gov/omim/603550)^N/S^ | HMX1^S^ | [MYH14](http://www.ncbi.nlm.nih.gov/omim/608568)^N/S^ | [POU4F3](http://www.ncbi.nlm.nih.gov/omim/602460)^N^ | [SOX10](http://www.ncbi.nlm.nih.gov/omim/602229)^S^ | RNR1^N^ |
| [COL4A6](http://www.omim.org/entry/303631)^N/S^ | FGF3^S^ | HOXA2^S^ | [MYH9](http://www.ncbi.nlm.nih.gov/omim/160775)^N/S^ | PROK2^S^ | [STRC](http://www.ncbi.nlm.nih.gov/omim/606440)^N^ | TS1^S^ |
| CO1^S^ | miR-96^N^ | miR-182^N^ | miR-183^S^ |  |  |  |

^N^ Genes for non-syndromic hearing loss; ^S^ Genes for syndromic hearing loss

| **Laboratory** | **Panel** | **Number of targeted genes** | **Ethnicity Tested** | **Whether *NLRP3* is included** | **References** |
| --- | --- | --- | --- | --- | --- |
| Seoul National University Bundang Hospital &Seoul National University Hospital | TRS-204 | 204 | Korean | No | [[5](#_ENREF_5)] |
| Otolaryngology & Renal  Research Labs, University of Iowa Molecular | OtoSCOPE | 54/59/66 | Mixed | No | [[6](#_ENREF_6)] |
| Department of Otolaryngology, Eye & ENT Hospital, Fudan University | Human Deafness Panel oto-DA3 | 131 | Chinese | No | [[3](#_ENREF_3)] |
| Ear Institute, Shanghai Jiaotong University School of Medicine | Unnamed | 79 | Chinese | No | [[8](#_ENREF_8)] |
| Institute of Human Genetics, Julius-Maximilians-Universität Würzburg | Unnamed | 80 or 129 | European | No | [[7](#_ENREF_7)] |
| Laboratory of Auditory Disorders, National Institute of Sensory Organs, National Hospital Organization Tokyo Medical Center | Unnamed | 84 | Japanese | No | [[4](#_ENREF_4)] |

**Supplementary Table S2.** Representative targeted NGS panels for deafness in previous reports
